# Cryoglobuline et facteurs associés chez le patient porteur de l'anticorps du virus de l'hépatite C dans un pays à ressources limitées

**DOI:** 10.11604/pamj.2019.33.169.19162

**Published:** 2019-07-04

**Authors:** Servais Albert Fiacre Bagnaka Eloumou, Jean Pierre Nda Mefo'o, Winnie Tatiana Bekolo Nga, Gabin Ulrich Kenfack, Linus Yakana, Agnès Malongue, Cecile Okalla, Mathurin Kowo, Firmin Ankoune Andoulo, Christian Tzeuton, Marie Solange Doualla Bidja, Henry Luma Namme, Dieudonne Adiogo, Dominique Noah Noah

**Affiliations:** 1 Service de Médecine Interne, Hôpital Général de Douala, Douala, Cameroun; 2 Faculté de Médecine et des Sciences Pharmaceutiques, Université de Douala, Douala, Cameroun; 3 Service de Biologie, Hôpital Général de Douala, Douala, Cameroun; 4 Faculté de Médecine et des Sciences Biomédicales, Université de Yaoundé I, Cameroun; 5 Centre Médical des Capucines, Douala, Cameroun

**Keywords:** VHC, cryoglobuline, ressources limitées, méthode de Biuret, Cameroun, Hepatitis C virus (HCV), cryoglobulin, limited resources, Biuret method, Cameroon

## Abstract

**Introduction:**

Le virus de l'hépatite C (VHC) a plusieurs manifestations extra hépatiques parmi lesquelles la cryoglubulinémie. La cryoglobulinémie se définit par la présence anormale dans le sang d'une ou plusieurs protéines (cryoglobuline) pouvant précipiter au froid.

**Méthodes:**

Nous avons mené une étude transversale et analytique dans le service du laboratoire de biologie et l'unité d'hépatologie de l'Hôpital Général de Douala (HGD) pendant une durée de 6 mois. Etaient inclus dans le travail tous les patients acceptant de participer et porteurs d'un anticorps anti VHC avec ou sans traitement. Les cryoglobulines étaient recherchées par la méthode de Biuret et la classification était réalisée par une immunoélectrophorèse de Brouet. Une analyse multivariée a été réalisée, des facteurs de confusion tels que l'âge, le sexe et la durée après dépistage du VHC ont été ajustés.

**Résultats:**

Nous avons inclus 116 patients. L'âge moyen était de 58,47 ± 9,95 ans. Le sexe masculin représentait 50,86% des cas. L'arthralgie était présente dans 69,80% des cas. La cryoglubiline était présente chez 63,80% des cas. Apres ajustement, le sexe féminin (ORa =2,18; IC à 95% [0,97-4, 90]; p= 0,059), l'asthénie seule (ORa =2,45; IC à 95% [1,04-5,80]; p= 0,041), l'asthénie couplée à l'arthralgie (ORa =2,84; IC à 95% [1,13-7, 10]; p= 0,026) et la présence de l'ARN du VHC (ORa =2,84; IC à 95% [1,13-7, 10]; p= 0,028) étaient des facteurs indépendamment associés à la présence de cryoglobuline.

**Conclusion:**

La prévalence de la cryoglobubine est élevée chez les patients porteurs de l'Ac anti VHC à l'HGD. Elle est recherchée par les méthodes biologiques simples. La recherche de cryoglobuline chez les patients porteurs du VHC est essentielle dans un pays à ressources limitées.

## Abstract (en)

**Introduction:**

hepatitis C virus (HCV) has several extra-hepatic manifestations including cryoglubulinemia. Cryoglobulinemia is defined as the abnormal presence in the blood of one or several proteins (cryoglobulins) that can precipitate at low temperatures.

**Method:**

We conducted a cross-sectional analytical study in the Laboratory of Biology and in the Unit of Hepatology of the General Hospital in Douala (HGD) over a period of 6 months. All patients agreeing to participate to the study and with anti-hepatitis-C antibodies under treatment or not were enrolled. Cryoglobulins were detected using biuret method and the classification was performed using Brouet immunoelectrophoresis. A multivariate analysis was conducted, confounding factors such as age, sex and the length of time after Hepatitis C Virus screening were adjusted.

**Results:**

The study enrolled 116 patients. The average age of patients was 58.47±9.95 years. Male sex accounted for 50.86% of cases. Arthralgia was found in 69.80% of cases. Cryoglobulin was found in 63.80% of patients. After adjustment, female sex (OR =2.18; CI 95% [0,97-4,90]; p= 0.059), asthenia alone (OR =2.45;CI 95% [1,04-5,80]; p= 0.041), asthenia combined with arthralgia (OR =2.84;CI 95% [1,13-7, 10]; p= 0.026) and the presence of HCV RNA (OR =2.84;CI 95% [1,13-7,10]; p= 0.028) were factors independently associated with the presence of cryoglobulin.

**Conclusion:**

The prevalence of cryoglobubin is high in patients with anti-hepatitis-C antibodies at the HGD. Simple biological methods are used to detect it. Cryoglobulin test in patients with HCV is essential in resource-limited countries.

## Introduction

L'infection par le virus de l'Hépatite C (VHC) est un problème de santé publique entraînant une augmentation de la morbidité et de la mortalité dans le monde [1]. Selon l'Organisation Mondiale de la Santé (OMS) en 2015 environ 71 millions de personnes dans le monde étaient porteuses chroniques du VHC soit une prévalence de 1% [2]. Petruzziello *et al* estiment la prévalence à 2,5% soit 177,5 millions d'adultes infectés par le VHC [1]. En Amérique, en Europe, et en Afrique la prévalence moyenne du VHC est respectivement de 1,3%, 2,5% et 2,9% [1]. L'Egypte a la prévalence la plus élevées dans le monde avec 1,7% [1]. Au Cameroun la prévalence est estimée à 6,5% [3]. L'évolution silencieuse de la maladie, généralement asymptomatique et la fréquence élevée de passage à la chronicité expliquent l'existence d'un grand réservoir de sujets infectés [4]. Le VHC est associé à plusieurs manifestations extra hépatiques qui sont par ordre de fréquence croissant: le lymphome, les maladies systémiques et la cryoglobulinémie [5]. La cryoglobulinémie se définit par la présence anormale dans le sang d'une ou plusieurs protéines (cryoglobulines) qui peuvent précipiter au froid [6]. Les symptômes qu'elles provoquent sont inconstants et variés, dus à l' hyperviscosité du sang dans les extrémités, ou en raison de dépôt de complexes immuns (CI) le long de la paroi des vaisseaux à l'intérieur de la peau, des articulations, des reins et/ou du système nerveux périphérique [7,8]. Ceci entrainant plusieurs complications telles que le purpura vasculaire, arthralgie, atteintes neurologiques, et même néphropathies glomérulaires [9]. Damasco *et al* en 2001 notent que le sérum des patients porteurs d'une cryoglobulinémie contient des anticorps (Ac) anti-VHC dans 87 à 100% des cas, et de l'Acide Ribonucléique messager (ARNm) codant le VHC dans 71 à 100% des cas [10]. En Europe, dans le bassin méditerranéen, et aux Etats Unis d'Amérique, la prévalence des sujets positifs au VHC porteurs des cryoglobuline mixte (II et III) était respectivement 40-90%, 90%, et 60% [11]. En Afrique plus précisément en Egypte la prévalence d'une cryoglobulinémie mixte chez les patients atteints d'une infection chronique par le VHC est de 40% [12]. Les techniques de cryoprécipitation à 4^o^C, le dosage par Cryocrite et certaines techniques colorimétriques telles que celle de Biuret inverse, celle au rouge de pyrogallol et l'analyse qualitative par immunoélectrophorèse (IEP) et l'Immunofixation (IF) sont généralement utilisées pour la mise en évidence des cryogloblulines [13]. Grâce à L'évolution des procédures de diagnostic du VHC et des avancées dans la thérapie un regain d'intérêt pour les cryogloblulines a été observé [9]. De plus les meilleures connaissances de la physiopathologie du VHC dans la genèse des cryogloblulines, les stratégies thérapeutiques ont beaucoup évoluées pour la prise en charge des patients présentant des cryoglobulinémies [9]. Au regard de la prévalence élevée du VHC au Cameroun, des complications du VHC et des fréquences de plus en plus élevées des manifestations extra hépatiques parmi lesquelles la cryoglobulinémie, au vu du peu de données sur le sujet dans notre contexte, nous nous proposons de rechercher les cryogloblulines et les facteurs associés chez personnes porteurs de l'Ac anti VHC par des méthodes de recherche simples telles que le Biuret et l'immunoélectrophorèse de Brouet.

## Méthodes

**Type, cadre et période de l'étude**: il s'agissait d'une étude transversale et analytique qui s'est déroulée à l'Hôpital Général de Douala. Cet hôpital à une capacité de 320 lits et possède de nombreux services et unités parmi lesquels le service de laboratoire de biologie clinique et l'unité d'hépato-gastro-entérologie. Ce travail s'est déroulé pendant six (06) mois du premier 1^er^ décembre 2016 au 31 mai 2017.

**Collecte des données**: la population cible était celle des patients porteurs de l'Ac anti HVC âgés de plus de 18 ans à l'HGD. Toutes personnes ayant les Ac anti VHC ou l'ARN du VHC préalablement diagnostiqué et documenté, acceptant de participer à l'étude ont été inclus dans l'étude. N'étaient pas inclus les patients refusant de participer à l'étude, les patients sous traitement par un anticoagulant et les dossiers incomplets. La taille minimale de l'échantillon calculé à l'aide de la formule de Lorentz était de 138 personnes. L'échantillonnage était consécutif. Le recrutement des patients s'est effectué par la consultation des registres de l'unité d'hépato-gastro-entérologie où les numéros de téléphones des patients ont été collectés. Les patients éligibles à notre étude contactés puis reçus sur rendez-vous selon leur disponibilité entre 8-12h à jeun les jours ouvrables. Les patients étaient reçus en consultation puis une explication verbale leur était faite concernant la cryoglobulinémie. Puis des fiches d'information et de consentement éclairé étaient remises au patient pour lecture pendant un temps qui leur était approprié. Nous laissions libre choix au patient de décider de sa participation à l'étude. Les données socio démographiques (âges, sexes, région d'origine, comorbidité) des patients étaient obtenues sur une fiche technique préétablie et remplie par le patient; les données cliniques (douleurs articulaires et musculaires), les données histologiques (score de fibrose), biologiques (numération formule sanguine (NFS), Alanine Aminotransférase (ALAT), Aspartate Aminotransférase (ASAT), créatinine, glycémie à jeun, cholestérol totale, triglycérides, hight density lipoproteins (HDL) et virologique (ARN VHC, génotype VHC) ont été obtenues à partir des dossiers médicaux des patients.

**Procédure biologique**: les patients inclus dans l'étude étaient soumis à un prélèvement de 10 ml de sang dans un tube sec avec des aiguilles préchauffées dans une étuve à 37°C. Le prélèvement placé dans un thermos pour transport, a été placé dans un bain-marie (Memmert^®^) à +37°Celsius jusqu'à rétraction du caillot (2 heures au moins, 24 heures au plus). En l'absence du thermos, les tubes ont été immédiatement placés dans un bain-marie. L'analyse des cryoglobulines a débuté par la centrifugation de nos échantillons en 15 min à 3500 t/min, dans des plots préchauffés à +37°C dans une centrifugeuse thermostatée (Uninversal 320R, fabricant: Hettich in Germany). Deux millilitres de sérum ont été décantés immédiatement dans un tube en verre fin long et étroit, pour bien visualiser le précipité. Ces tubes ont été bouchés et placés au réfrigérateur à +4°C, pendant au moins 10 jours et même jusqu'à un mois. Si les précipités étaient présents (petits grains ou flocons, mie de pain, aspect de gel au fond du tube ou d'un gel occupant tout le volume du sérum), les tubes étaient centrifugés à +4°C pendant 15 minutes à 3500 tours /minute et le surnageant éliminé. Le précipité était lavé 3 fois à +4°C dans 1 ml de solution de NaOH maintenu à +4°C, pour éliminer les autres protéines sériques. Dans le précipité pur, 200μl de solution de NaOH étaient ajoutées puis portées au bain-marie à +37°C pendant 30 minutes à 1 heure pour resolubilisation, puis la solution obtenue était mise dans les micro-tubes et portée à -20°c pour un usage ultérieur. Avant utilisation les tubes étaient placés à 37°C dans un bain-marie pour être décongelés. Le dosage à base de protéines totales mono liquide (TP : 21255141, N°lot: 1618 fabricant: Inmesco GmbH, in Germany) des cryogloblulines était effectué par la méthode de Biuret [14] dans un spectrophotomètre visuel (Biomerieux, N° série:60VB0357) et la classification faite par la méthode d'immunoélectrophorèse de Brouet [15].

**Définition des termes opérationnels**: la consommation d'alcool était considérée comme moindre lorsque l'index éthylique était inférieur à 50 g /j et importante lorsqu'il était supérieur à 50 g / jour. Était considéré comme hypertendu ou diabétique tout patient dont le diagnostic a été confirmé par un cardiologue ou l'endocrinologue dans le dossier du malade. Les manifestations rhumatologiques étaient subdivisées en trois : atteintes articulaires qui concernaient celles des mains, des poignets, des orteils, des genoux et autres (épaule, hanche, coude) ; des douleurs musculaires et enfin la fatigue. Au cours de cette étude était considéré comme patient sans ARN du VHC, des patients qui avaient une infection chronique au VHC guéris par un traitement d'une part, et des patients possédant l'Ac anti VHC chez qui l'ARN VHC n'avait jamais été retrouvé d'autre part. Les patients avec ARN, étaient ceux chez qui un ARN du VHC était présent sans ou en cours de traitement. La charge virale du VHC était considérée comme élevée lorsqu'elle était supérieure ou égale à 400 000 UI/ml et faible lorsqu'elle était inférieure à 400 000 UI/ml [16]. La fibrose était corrélée au score de METAVIR, qui permettait de classer la fibrose hépatique en cinq stades: F0 (absence de fibrose), F1 (fibrose portale sans septa), F2(fibrose portale avec quelques septa), F3 (fibrose portale avec nombreux septa sans cirrhose), F4(cirrhose). Nous avons considéré comme : Fibrose significative : le score de fibrose < F2 et Fibrose non significative: le score de fibrose < F2.

**Analyse statistique**: les logiciels: Microsoft Word 2010, Excel 2010, SSPS (Statistical Package for Social Sciences) version 20.0 ont été utilisés pour le traitement et l'analyse des données. Les statistiques descriptives ont été appliquées pour ordonner, classer et condenser les variables. Les variables qualitatives ont été exprimées sous forme de fréquence et effectifs. Les variables quantitatives ont été exprimées en moyenne ± écart-type. Le test de Khi 2 a été utilisé pour rechercher l'association entre les groupes. Les facteurs associés ont été déterminés à l'aide d'un test de régression logistique pour une analyse univariée et multivariée. Si la covariable ne suivait pas une distribution normale, un test du Khi2 était utilisé pour vérifier l'association. Au cours de l'analyse multivariée, des facteurs de confusion tels que l'âge, le sexe, la durée après dépistage du VHC ont été ajustés. La force de l'association a été délivrée sous la forme d'un odds-ratio avec l'IC à 95%. Les valeurs p ≤ 0,2 (après ajustement) et <0,05 ont été considéré comme statistiquement significatives.

**Considérations éthiques**: la réalisation de ce travail a été approuvé par le comité d'éthique institutionnel pour la recherche en santé humaine de l'université de Douala sous le numéro CEI-Udo/722/01/2017/M et les autorités de l'HGD sous le numéro 048 AR/MINSANTE/HGD/DM/02/17. Nous avons travaillé en conformité avec les lois bioéthiques, la loi informatique et de liberté, ainsi qu'en accord avec les bonnes pratiques cliniques et la déclaration d'Helsinki.

## Résultats

**Figure 1:**
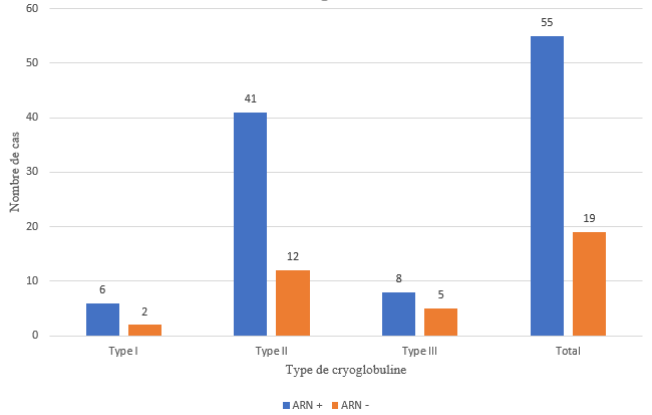
Distribution des cryoglobulines dans la population d'étude

Deux cent cinquante-cinq dossiers des patients positifs au VHC ont été répertoriés au cours cette étude. Nous avons inclus un total de 116 patients soit 45,45%. Le Tableau 1 décrit les caractéristiques générales de la population. L'âge moyen était de 58,47 ± 9,95 ans. Le sexe masculin représentait 50,86% des cas (n=59). La consommation d'alcool était observée 34,48% des cas (n=40) et elle était supérieure à 50 g/j chez 32,50% des cas (n=13). La présence des symptômes cliniques était de 83,30% (n=97) et l'arthralgie était présente dans 69,80% des cas (n=81). Les patients n'ayant reçu aucun traitement contre le VHC représentaient 57,55% des cas (n=61). Un ARN du VHC était présent chez 66,38% des patients (n=77). Une charge virale VHC élevée représentait 51,10% des cas (n=45). Le génotype 1 était présent chez 49,38% des cas (n=40). La fibrose était significative chez 73,30% des patients (n=44). La Figure 1 présente la proportion de la cryoglobulinémie dans la population de l'étude. Nous constatons que la cryoglobulinémie était présente chez 63,80% des cas (n=74). Trois types de cryogloblulines ont été observées à savoir le type I, II et III avec respectivement 6,89%, 45,68% et 11,21%. La cryoglobuline mixte (CM) (Type I et II) était présente dans une proportion de 52,57% de cas. Les patients avec la présence de l'ARN du VHC avaient plus de cryoglobuline que ceux n'ayant pas l'ARN 47,42% versus 16,38% (p=0,016). Les patients de sexe masculin présentaient moins de cryoglobuline que ceux de sexe féminin (57,60% versus 70,20%, p=0,031). Le Tableau 2 présente la relation entre les différentes variables et la présence ou non de cryoglobuline. La cryoglobuline est beaucoup plus présente chez les patients de sexe féminin avec 70,20% versus 57,60% chez les patients de sexe masculin (p=0,031). La présence de l'ARN du VHC a un lien avec la présence de cryoglobuline avec 74,40% versus 48,70% pour l'absence de l'ARN du VHC. La charge virale qu'elle soit élevée ou basse et le génotype n'avaient aucun lien avec la présence de cryoglobuline. Le Tableau 3 met en évidence les facteurs indépendants associés à la présence des cryoglobulines. Apres ajustement, le sexe féminin (ORa =2,18;IC à 95% [0,97-4,90]; p= 0,059), l'asthénie seule (ORa =2,45;IC à 95% [1,04-5,80]; p= 0,041), l'asthénie couplé à l'arthralgie (ORa =2,84;IC à 95% [1,13-7,10]; p= 0,026) et enfin la présence de l'ARN du VHC (ORa =2,84;IC à 95% [1,13-7,10]; p= 0,028) étaient des facteurs indépendamment associés à la présence de cryoglobuline.

**Tableau 1:** Caractéristiques générales de la population d’étude

Variables	Effectifs
**Ages (années) (N= 116)**	58,47 ± 9,95
**Co morbidités (N= 116)**	
Diabète	23(19,80%)
Co infection VHB	2(1,70%)
Co infection VIH	3(2,60%)
Alcool	
<50 g/j	27(67,50%)
≥50 g/j	13(32,50%)
**Statut thérapeutique (N= 106)**	
VHC traité	29(27,36%)
VHC non traité	61(57,55%)
VHC en cours de traitement	16(15,09%)
**Symptômes cliniques (N= 116)**	
Non	19(16,70%)
Oui	97(83,30%)
Arthralgie	81(69,80%)
Myalgie	78(67,20%)
Asthénie	49(42,60%)
Arthralgie + Myalgie	66(56,90%)
Arthralgie + Asthénie	39(33,60%)
Asthénie + Myalgie	40(34,50%)
Arthralgie + Myalgie + Asthénie	38(32,80%)
**Biologie**	
Présence ARN VHC (N= 116)	
Oui	77(66,38%)
Non	39(33,62%)
ALAT (UI/l)	47,91 ± 51,76
ASAT (UI/l)	48 ± 43,57
Glycémie à jeun (g/l)	0,95 ± 0,28
Créatinine (μmol/l)	86,39±47,45
HDL (g/l)	0,57±0,20
Cholestérol total, (g/l)	1,76±0,31
Triglycéride (g/l)	1,08±0,81
**ARN VHC (UI/ml) ( N= 85)**	
<400 000	40(48,90%)
≥400 000	45(51,10%)
**Génotypes (N= 81)**	
1	40(49,38%)
2	22(27,16%)
4	19(23,46%)
**Score de Fibrose (N=60)**	
<F2	16(26,70%)
≥F2	44(73,30%)

**Tableau 2:** Relation entre les différentes variables et la présence ou non de cryoglobuline

Données	CG positif n(%)	CG négatif n(%)	P value
**Ages (années)**			
≤ 61	37(63,80)	21(36,20)	1
>61	37(63,60)	21(36,20)	
**Sexe**			
Masculin	34(57,60)	25(42,40)	**0,031**
Féminin	40(70,20)	17(29,80)	
**Diabète**			
Non	58(62,40)	35(37,60)	0,521
Oui	16(69,60)	7(30,40)	
**Consommation alcool (g/j)**			
<50	17(63,00)	10(37,00)	0,382
≥50	10(79,90)	3(23,10)	
**Arthralgie**			
Non	67(63,80)	38(36,20)	0,991
Oui	7(63,60)	4(36,40)	
**Myalgie**			
Non	71(64,50)	39(35,50)	0,476
Oui	3(50,00)	3(50,00)	
**Asthénie +Myalgie + Arthralgie**			
Non	45(57,70)	33(42,30)	0,053
Oui	29(76,30)	9(23,70)	
**Statut ARN du VHC**			
ARN(-)	19(48,70)	20(51,30)	**0,018**
ARN(+)	55(74,40)	22(28,60)	
**Charge virale VHC (UI/ml)**			
< 400000	22(55,00)	18(45,00)	0,08
≥400000	33(73,30)	12(26,70)	
**Fibrose**			
<F2	9(24,30)	7(30,40)	0,60
≥F2	28(75,70)	16(69,60)	
**Génotype VHC**			
1	23(57,50)	17(42,50)	ref
2	15(68,20)	7(31,80)	0,16
4	17(68,00)	8(32,00)	0,20

CG: cryoglobuline

**Tableau 3:** Facteurs indépendants associés à la présence des cryoglobulines dans la population d’étude

Données	CG positif		CG négatif		Analyse multivariée	
	**n**	**(%)**	**n**	**(%)**	**ORa [IC95%]**	**P Value**
**Sexes**						
masculin	34	(57,60)	25	(42,40)	1	
féminin	40	(70,20)	17	(29,80)	2,18 [0,97-4,90]	**0,059^$^**
**Statut ARN du VHC**						
ARN(-)	19	(48,70)	20	(51,30)	1	
ARN(+)	55	(71,40)	22	(28,60)	2,56 [1,11-5,91]	0,028*
**Asthénie**						
non	37	(56,10)	29	(43,90)	1	
oui	36	(73,50)	13	(26,50)	2,45 [1,04-5,80]	0,041*
**Asthénie+ arthralgie**						
non	44	(57,10)	33	(42,90)	1	
oui	30	(76,90)	9	(23,10)	2,84 [1,13-7,10]	0,026*

CG: cryoglobuline

## Discussion

Au terme de ce travail, il ressort que la proportion de cryoglobuline dans cette population est de 63,80%. Trois types de cryoglobulines étaient observées dans la population d'étude (le type I, II et III). La cryoglobuline mixte était présente dans 52,57%. Le sexe féminin, l'asthénie de façon isolé et associé à l'arthralgie, la présence de l'ARN du VHC étaient les facteurs indépendamment associés à la présence des cryoglobulines. Les limites de ce travail étaient de plusieurs ordres. Il s'agissait de la taille réduite de l'échantillon, l'absence de plateau technique pour le typage des immunoglobulines par immunofixation, le cout des autres examens pour l'évaluation du VHC. Mais cette étude a le mérite d'être la première à se pencher sur la problématique de la cryoglobulinémie chez les porteurs de l'Ac anti-VHC au Cameroun. L'âge moyen de la population dans ce travail est proche celui des autres séries au Cameroun [17,18]. La prédominance était masculine dans ce travail avec 50,86% des cas. Elle semble être à l'opposé des autres travaux au Cameroun qui retrouvait une prédominance féminine à 53% d'une part et d'autre part 58,6% [17,18]. La sélection des patients dans ce travail s'est faite dans un mode prospectif. Le mode de sélection des patients en rétrospectif dans les autres travaux peut expliquer cette différence. Il faut noter que le travail de Eloumou *et al* n'avait sélectionné que les patients porteurs des génotypes 1 et 4 et qui recevaient un traitement spécifique. La douleur articulaire était retrouvée dans 69,80% dans ce travail ce qui contraste avec les résultats de la série de Luma *et al* [17] qui la retrouvait 9% des cas en 2016. Luma *et al* avait recherché les informations dans les dossiers en rétrospectif. La recherche de l'information telle que les douleurs articulaires n'est pas mentionnée de façon systématique dans les dossiers des patients. La cryoglubuline était présente à 63,80% de cas dans ce travail. Ces résultats se rapprochent de ceux retrouvés par Saadoum *et al*, Charles *et al* et De Gragnagni *et al* qui ont respectivement retrouvés en 2007, 2009 et 2014 des prévalences de 65%, 60% et 66,67% [19-21]. Ils s'éloignent de ceux retrouvés en Egypte en 2015 qui était de 40% [12]. La prévalence de la cryoglobulinemie est fortement liée à la prévalence du VHC [7]. Il faut aussi souligner que dans le travail actuel nous avons travaillé sur tous les types de cryoglobulines. Contrairement en Egypte, seule la cryoglobulinémie mixte avait été prise en compte [12]. Nous avons retrouvé la cryoglobuline mixte dans 52,57% des cas ce qui se rapproche des résultats de la série de El-Bendary *et al* en Egypte en 2015 [12]. Le sexe féminin, la présence de l'ARN du VHC et des certains symptômes comme l'asthénie seul ou associée à une arthralgie étaient des facteurs indépendants associés à la présence de cryoglobuline dans ce travail. En Italie en 2000, Cicardi *et al* retrouvait que le sexe féminin était associé à la présence de cryoglobuline [22]. L'association entre cryoglobuline et la gravité de la maladie du foie dans l'hépatite C demeurent une question controversée. Dans une méta-analyse, Kayali *et al* retrouvaient une association significative entre la cirrhose et la cryoglobuline après ajustement pour l'âge, le sexe et la durée d'infection [23]. Il faut noter que la recherche de cryoglobuline est très complexe et l'absence de standardisation des méthodes diagnostic reste un véritable problème [24].

## Conclusion

Il ressort de ce travail que la cryoglobuline est présente chez les patients porteurs du VHC avec ou sans la présence de l'ARN. Cette cryoglobuline est dans la majorité des cas mixte. La cryoglobuline est associée de façon indépendante au sexe féminin, à la présence de l'ARN et à l'asthénie isolée ou associée à l'arthralgie. Ce travail nous incite à rechercher de façon systématique la cryoglobuline dans les pays à ressources limités par une méthode simple qui est celle de Biuret.

### Etat des connaissances actuelles sur le sujet

- La cryoglobulinemie est une manifestation extra-hépatique fréquente;
- Les manifestations sont de l'ordre d'atteintes cutanées, articulaires, rénales et du système nerveux périphérique.

### Contribution de notre étude à la connaissance

- La prévalence de la cryoglobuline chez les malades porteurs du virus de l'hépatite C en Afrique sub-saharienne;
- Le type de cryoglobuline le plus fréquent dans notre milieu;
- La technique de recherche de la cryoglobuline qui est accessible et peu couteuse.

## Conflits d’intérêts

Les auteurs ne déclarent aucun conflit d’intérêts.
